# A Review of Polysiloxanes in Terms of Their Application in Explosives

**DOI:** 10.3390/polym13071080

**Published:** 2021-03-29

**Authors:** Karol Zalewski, Zbigniew Chyłek, Waldemar A. Trzciński

**Affiliations:** Institute of Chemistry, Military University of Technology, 00-908 Warsaw, Poland; zbigniew.chylek@wat.edu.pl (Z.C.); waldemar.trzcinski@wat.edu.pl (W.A.T.)

**Keywords:** polysiloxanes, explosives, PBX

## Abstract

Polysiloxanes are reviewed for their properties depending on the functionalization of a silicon–oxygen backbone chain. Next, the properties were referred to the requirements that polymers used in plastic/polymer-bonded explosive (PBX)-type explosives must meet. Finally, the current state and prospects for the implementation of polysiloxanes in plastic/polymer-bonded explosive (PBX) formulations are presented.

## 1. Introduction

The problem of the excessive sensitivity of explosives has troubled people since the time of nitroglycerin, invented by Ascanio Sobrero. The answer to frequent accidents associated with it was dynamite, developed by Alfred Nobel [1]. This invention made work with nitroglycerin easier and safer. The problem of excessive sensitivity, however, not only concerned nitroglycerin. The military high explosives used from the beginning of the 20th century around the world also had to have sufficiently low sensitivity. This problem was solved by phlegmatizing explosives with natural polymers, waxes and paraffins.

The development of polymer chemistry in the second half of the 20th century allowed the use of new synthetic polymers for phlegmatization. This combination has created a completely new branch of explosive compositions called PBX (plastic/polymer-bonded explosive). The first attempts to produce mixtures containing crystalline explosives and polymers were carried out by Frankel and Carleton [2]. PBX-type compositions are characterized by greater stability and greater resistance to atmospheric agents, higher detonation parameters and greater safety compared to mixtures with natural substances. In the literature, there is information on many interesting polymers acting as a binder in PBXs [2,3,4,5,6,7,8,9]. The most commonly used are hydroxyl-terminated polybutadiene (HTPB), fluoropolymers: Teflon (polytetrafluoroethylene—PTFE), Kel F (polychlorotrifluoroethylene—PCTFE), Viton A (copolymer of vinylidene fluoride and hexafluoropropylene—VDF-co-HFP), styrene–butadiene rubber (SBR) and thermoplastic elastomers (TPE). The TPE group includes thermoplastic polyurethanes (TPU, e.g., Estane), thermoplastic polyolefins (TPO, e.g., EVA copolymers), thermoplastic polyamides (TPA), thermoplastic styrene block copolymers (TPS) and thermoplastic polyesters (TPC, e.g., HyTemp). These polymers are a group of energetically inert binders; they do not have explosophore groups in their structure. Energetic polymers, such as poly(3-nitroxymethyl-3-methyloxetane) (polyNIMMO) and poly(glycidyl azide) (GAP), also play an important role in modern PBX compositions [5,6,7,8,10].

Due to their unique properties, polysiloxanes in the forms of greases, oils and rubbers have been used in many industries. Studies have shown that silicones can be an interesting group of polymers in PBX-type explosives.

## 2. Physicochemical Properties of Polysiloxanes Depending on the Functionalization of the Silicon–Oxygen Backbone

Polysiloxanes are organosilicon polymers whose chains are composed of alternating silicon and oxygen atoms and side groups, usually organic, attached to silicon atoms. Polysiloxanes show very interesting properties resulting from their specific structure. Different values of O-Si-O and Si-O-Si angles, as well as the lack of substituents on oxygen atoms, imply high chain flexibility. In polysiloxanes with small side groups (e.g., methyl, ethyl) the energy barrier of rotation around the bond Si-O is very small. In addition, the silicon–oxygen bond is about 50% ionic and partially double (interaction of free oxygen electron pairs with the empty silicon orbital) [11]. The literature provides information on many interesting properties [12,13], such as low surface tension, hydrophobicity, good surface wettability, low glass transition temperature, high compressibility and damping properties, liquid properties (even for high molecular weights), low boiling and freezing points, low dependence of physical properties on temperature, low reactivity, low toxicity and flammability and low harmfulness to the natural environment.

**Figure 1:**
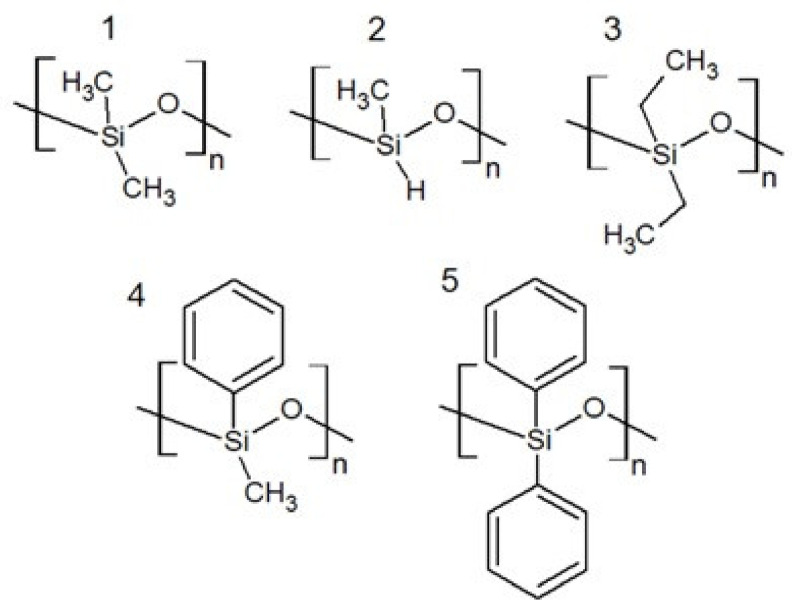
Examples of polysiloxanes with simple side groups; (**1**) PDMS, (**2**) polymethylhydrogensiloxane (PMHS), (**3**) polydiethylsiloxane (PDES), (**4**) polyphenylmethylsiloxane (PMPS), (**5**) polydiphenylsiloxane (PDPS).

The most popular and best-studied polysiloxane is polydimethylsiloxane (PDMS, Figure 1). For the purposes of this paper, it is assumed that PDMS is the basic polysiloxane to which other organosilicon polymers will be related. The properties of polydimethylsiloxane change as the average molecular weight changes (Table 1). At 25 °C, dynamic viscosity is in the range of from 3 to 500,000 mPa·s, depending on the average molecular weight of the polymer. The density and surface tension of PDMS do not depend so strongly on the average molecular weight. At 25 °C, the density ranges from 0.90 to 0.97 g/cm^3^, while the surface tension ranges from 19.3 to 21.5 mN/m. Polydimethylsiloxane has one of the lowest glass transition temperatures of −123 °C [11].

Modification of the side groups or groups at the ends of the polydimethylsiloxane chain significantly affects its properties. The lateral methyl group can be replaced by, among others, a hydrogen atom; longer-chain alkyl groups; aryl groups, vinyl groups; or their derivatives containing halogen atoms, amine, hydroxyl, thiol or epoxy groups [15].

The replacement of one methyl group with a hydrogen atom in the PDMS structural unit results in the formation of polymethylhydrogensiloxane (PMHS, Figure 1), which implies a decrease in the glass transition temperature to the level of −138 °C [11]. The presence of the hydrogen atom connected directly to the silicon atom ensures the reactivity of the polysiloxane to vinyl groups [16], which is used to modify polysiloxanes by hydrosilylation [17]. Similarly, functionalization of the polysiloxane chain with vinyl groups paves the way for many cross-linking methods.

Polysiloxanes with functional groups in the form of hydrocarbon chains with a number of carbon atoms greater or equal to three have lower chain flexibility, which implies higher glass transition temperatures [11]. An interesting case is polydiethylsiloxane (PDES, Figure 1), which despite having a larger side group, has a lower glass transition temperature than PDMS of −139 °C [11]. The authors [11,16] also note the ability of long-chain side group polysiloxanes to form liquid crystalline phases.

As in the case of big alkyl groups, the large steric hindrance introduced by aromatic groups affects the flexibility of the siloxane chain, which implies high glass transition temperatures, as for polysiloxanes. The introduction of one phenyl group (polyphenylmethylsiloxane—PMPS, Figure 1) into the chain implies a glass transition temperature of −28 °C and a density in the range of 1.05 to 1.12 g/cm^3^ [11,18]. For polydiphenylsiloxane (PDPS, Figure 1), the glass transition temperature rises to 40 °C [11]. The presence of a phenyl group implies high free-surface energy of 33.2 mJ/m^2^ for PMPS (Table 2) [19].

**Figure 2:**
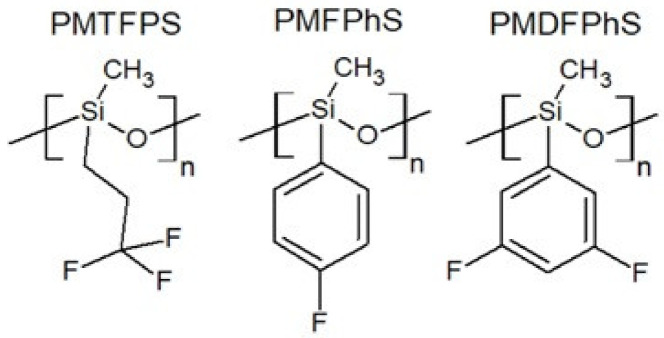
Examples of polysiloxanes with fluorinated side groups.

Polymethyl(3,3,3-trifluoropropyl)siloxane (PMTFPS) is an easily available and well-studied representative of fluorofunctional polysiloxanes. The presence of the fluorinated functional group has a significant impact on the properties of polysiloxane. It has a higher density of about 1.293 g/cm^3^ (21 °C) with an average molecular weight of 14,000 g/mol and a viscosity of about 13,000 mPa·s [20]. PDMS achieves a similar viscosity at an average molecular weight of 26,000 g/mol. The glass transition temperature of PMTFPS is −70 °C. The presence of trifluoropropyl groups also implies increased solubility in polar liquids. PMTFPS has interesting surface properties [21], which are compared to the properties of PDMS and fluorinated polyphenylmethylsiloxanes that are synthesized and characterized in paper [19] (Table 2). In paper [22], a synthesis method of fluorofunctional polysiloxane is proposed by means of the hydrosilylation of fluorinated allyl ethers. Structural formulas of fluorinated polysiloxanes are shown in Figure 2.

**Figure 3:**
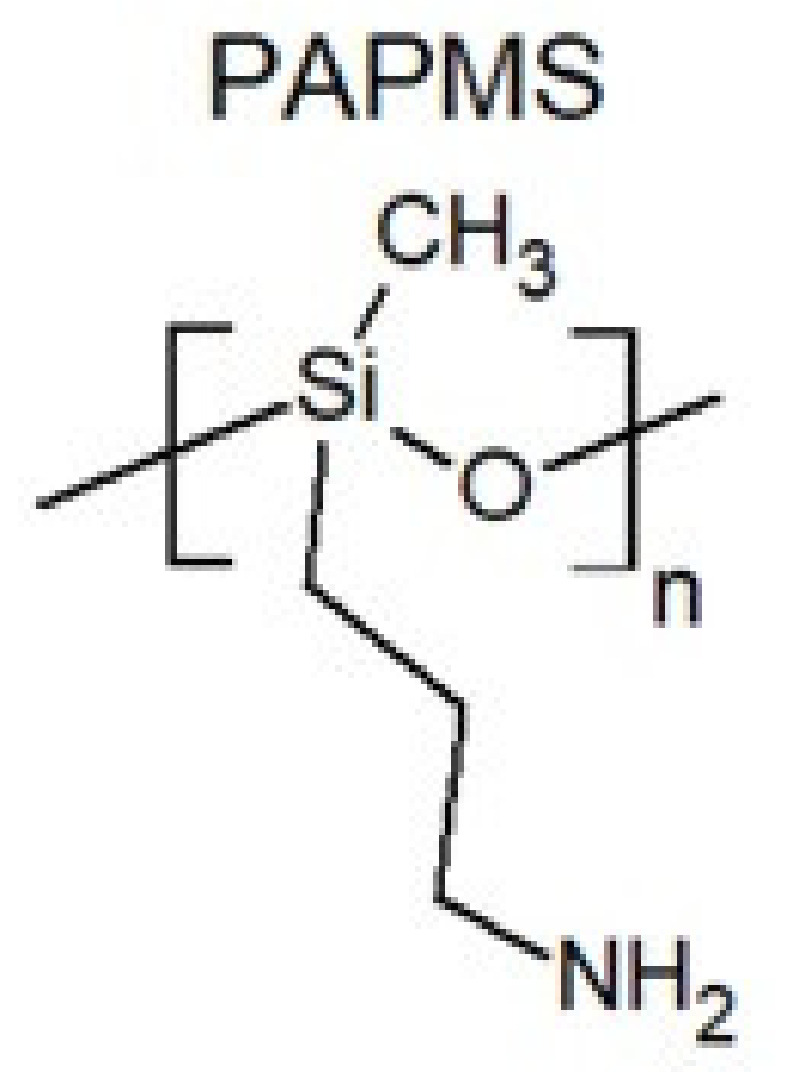
Poly(3-aminopropyl)methylsiloxane.

Polysiloxanes functionalized with aminoalkyl groups are used in the cosmetics and textile industries for their amphiphilic properties [23]. In paper [24], molecular dynamics of low molecular weight poly(3-aminopropyl)methylsiloxane (PAPMS, Figure 3) are investigated. The glass transition temperature of the tested polymer is −63 °C. The copolymer of polydimethylsiloxane and poly(3-aminopropyl)methylsiloxane with an average molecular weight of 4400 g/mol at 25 °C has a density of 0.96 g/cm^3^ and viscosity of 86.4 mPa·s [25]. Synthesis methods and the use of amino-functional polysiloxanes are presented in papers [23,26]. The authors in [26] synthesize and characterize amino-functional polysiloxanes containing lateral groups with different numbers of amino groups. The change in the amino value of the obtained polymers implies various lengths of polydimethylsiloxane fragments. The lengths of strongly hydrophobic polydimethylsiloxane segments affect the softening and smoothing ability of polymers. The presence of amino groups also allows reversible crosslinking with CO_2_ and CS_2_ [27].

The glass transition temperatures of the polysiloxanes discussed thus far are summarized in Table 3.

**Figure 4:**
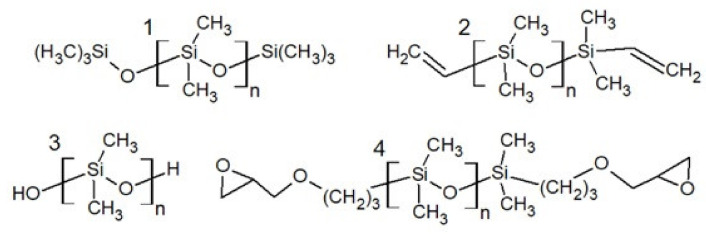
Polysiloxanes with different end groups: (**1**) trimethylsilyl, (**2**) vinyl, (**3**) hydroxyl, (**4**) epoxy.

On an industrial scale, PDMS can be obtained as a result of hydrolysis, e.g., dichlorodimethylsilane [14]. The resulting polydimethylsiloxane is terminated with hydroxyl groups. The PDMS chain ends were deactivated using a trimethylsilyl group introduced into the chain by the reaction of the hydroxyl group with chlorotrimethylsilane. In addition to the mentioned hydroxyl group, the reactive centers at the ends of the polysiloxane chain can comprise groups such as amino, epoxy, vinyl or alkoxysilane (Figure 4) [28,29,30,31]. Similarly to reactive side groups, they can be used in the curing process, e.g., the reaction of hydroxyl groups with isocyanates to form polyurethane. The influence of end groups on the physical properties of polysiloxanes can be observed in the example of PDMS terminated with hydroxyl groups. At the average molecular weight of approx. 550 g/mol, its viscosity is approx. 24 mPa·s [32]. PDMS with trimethylsilyl groups of similar average molecular weights has an eight times lower viscosity (Table 1).

**Figure 5:**
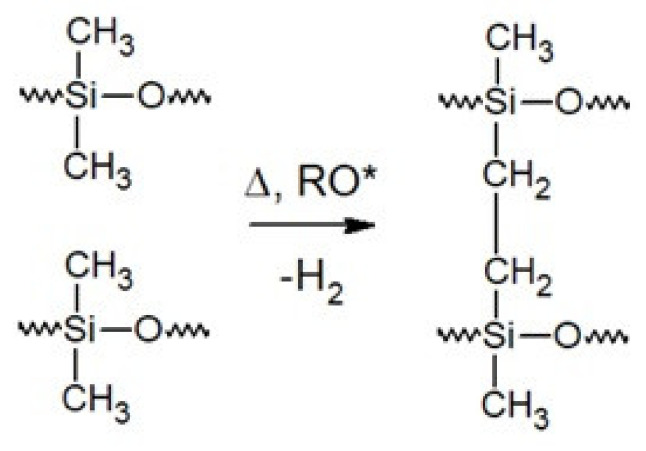
Curing with peroxides, RO*—radical.

The selection of appropriate side groups or groups at the ends of the polysiloxane chain enables the silicone curing process to be carried out by a variety of reactions. In the literature, silicone rubbers are divided into HTV rubbers (high-temperature vulcanizable) and RTV rubbers (room-temperature vulcanizable). There are three main curing mechanisms in HTV silicones: curing with peroxides (Figure 5), high-temperature-activated hydrosilylation and reactions of vinyl groups with mercaptoalkyl groups [12].

**Figure 6:**
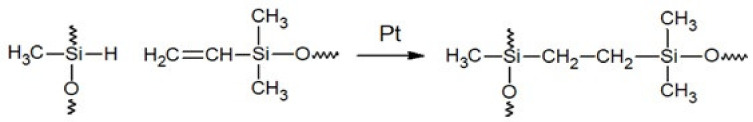
Curing via hydrosilylation.

In the case of RTV silicones, there are usually single-component condensation-type silicones and two-component addition-type silicones [12]. In the first case, crosslinking occurs after contact of the silicone with moisture from the air as a result of the hydrolysis of alkoxy or acetoxy groups. In addition-type silicones, the mechanism is based on a hydrosilylation reaction catalyzed by platinum compounds (Figure 6).

## 3. Implementation of Polysiloxanes in PBX Explosives—Current State and Perspectives

Studies of PBX compositions containing polysiloxanes are present in the literature [10,33,34,35,36]. The authors of paper [10] examine formulations containing FOX-7 and HMX (1,3,5,7-tetranitro-1,3,5,7-tetrazoctane) mixed with polyNIMMO and silicone grease. The compositions containing polysiloxane show friction sensitivity of greater than 360 N and impact sensitivity of greater than 20 J. The minimum normal force or impact energy at which the sample is initiated in at least one of six trials is considered to be the friction or impact sensitivity. A detonation velocity test is carried out for the most promising sample. The explosive with a density of 1.55 g/cm^3^ is placed in a 10 mm diameter PVC tube detonated at a velocity of 6960 ± 70 m/s. In paper [33], the authors draw attention to the interesting performance properties of linear cumulative charges with flexible formulations containing polysiloxanes in the form of RTV silicones. The prepared PBXs achieve detonation velocities ranging from 5620 to 7100 m/s (flat charge, 60 mm by 200 mm). The density of the compositions ranges from 1.28 to 1.40 g/cm^3^. The content of RTV silicones in the prepared formulations varies from 20 to 30%. Self-supporting compositions are allowed to eliminate the metal cover, which limits the negative impact of debris on the environment. In publication [34], the effect of aging time and temperature on the detonation velocity of the composition XTX-8003, consisting of 80% PETN and 20% Sylgard silicone rubber, is investigated. The composition is extruded into four square channels with widths of 2.03, 0.89, 0.51 and 0.38 mm in a polycarbonate block and then cured. The tested PBX detonates at a velocity of 7284 ± 25 m/s (before aging, largest channel). The authors propose an empirical Equation (1) that allows us to determine the service time, t (years), depending on the storage temperature, T (°C).

t = 147 ∗ exp(−0.0268 ∗ T)(1)

The authors of papers [35,36] point out the good phlegmatizing properties of silicones. Additionally, polysiloxanes show a neutral effect on the thermal stability of the obtained compositions. PBXs with polydimethylsiloxane are obtained by mixing 88% crystalline nitroamine with 12% silicone in a Brabender Plastograph for 90 min at 25 °C. Table 4 shows a comparison of the properties of compositions containing polysiloxanes with compositions containing popular polymeric binders and crystalline explosives. The normal force or impact energy value giving a 50% chance of sample initiation is considered as the friction or impact sensitivity. Detonation velocity tests are carried out using charges without casing with diameters of 16 mm and lengths of 200 mm. According to the authors, the use of polydimethylsiloxane can be the optimal solution between high detonation parameters and low sensitivity of the composition.

Analysis of the structure and properties of polymers included in the PBX explosives, as well as information contained in the literature [38,39,40,41,42], set requirements that polymers used in PBXs must meet. A desirable feature of the polymeric binder is, for example, a low glass transition temperature (preferably below −50 °C), which guarantees the invariability of the mechanical properties of the composition, even when used at low temperatures. Too high a glass transition temperature could lead to mechanical damage (cracking, breaking or crushing). A very important parameter associated with the production of explosive compositions is the viscosity of the binder. The viscosity should be easily modified by means of plasticizers by curing or by changing the temperature. Curing should be possible at temperatures significantly lower than the decomposition temperature of the crystalline explosive used in the composition. Appropriate mechanical properties are responsible for the durability of the composition, its reduced sensitivity and required properties (pressing granules, flexible composition, and plastic explosive susceptible to hand molding). The binder must also be chemically resistant, thus guaranteeing the isolation of the explosive crystals from atmospheric agents and chemicals that may affect the chemical stability of the PBX. In addition, during the production and storage of the composition, chemical reactions that may change the properties of PBX are not allowed. Another important feature that polymer binders must have is thermal stability. The polymer must not undergo any changes that may affect both the chemical and physical stability of PBX formulation at temperatures at which the given PBX will be stored and used. The binder must have a high affinity for the explosive crystals. In the binder–explosive system, efforts should be made to minimize the interfacial tension. Low interfacial tension implies high wettability of the crystal surface, which consequently improves the mechanical properties and physical stability of the formulation. An important criterion is also the economic issue. The used polymer should be as cheap as possible, easily accessible and manufactured by domestic suppliers, and its introduction to PBX production should be as easy as possible.

Polydimethosiloxane or its derivatives meet most of the requirements. The problem with polysiloxanes is the strongly inorganic nature of the backbone chain. Lack of significant affinity for the explosive crystals can lead to the low physical stability of the resulting compositions due to binder exudation and loss of solid phase (explosive crystals) during use.

In order to produce a stable PBX composition based on a polysiloxane binder, it is necessary to increase the interaction between the explosive crystals and the polymer. The first method may be to use organosilicon polymers with lateral groups that will create hydrogen bonds with the nitro groups of the explosive. The second method may be to use bonding agents that are commonly used in PBX and rocket propellants. The role of bonding agents is to adsorb on the surface of the explosive crystals and create chemical bonds with the binder [38]. Bonding agents such as aziridine derivatives, hydantoin derivatives, borate esters, isocyanurates, ferrocene derivatives and others are described [43,44].

**Figure 7:**
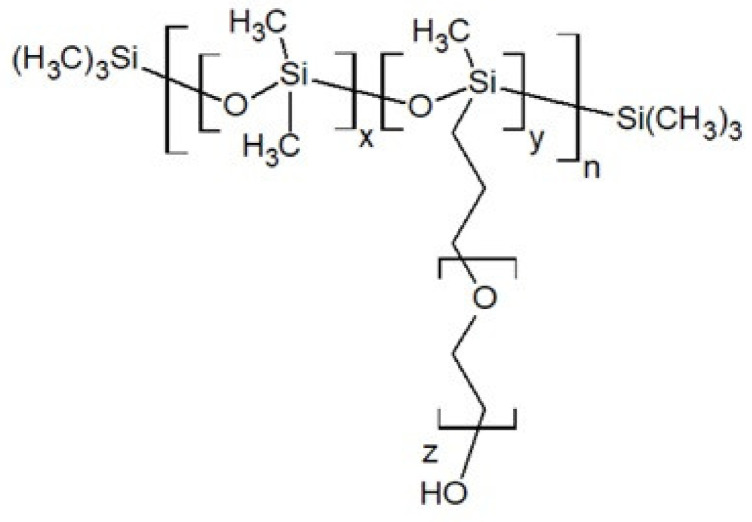
Polydimethylsiloxane/poly(ethylene oxide) graft copolymer.

Another important ingredient that is used in binder systems and that may help with the good implementation of polysiloxanes is a wetting agent (surfactant), e.g., lecithin [38]. Compounds of this type have in their structure polar and nonpolar parts that lower interfacial tension between solid and liquid ingredients of PBX or rocket propellants. In the silicone binder systems, it might be necessary to use siloxane surfactants, which, due to their unique properties, are used in polyurethane foams, the textile industry, cosmetics and paints [45]. Siloxane surfactants may have different chemical structures with different polar groups, such as polyoxyethylene (nonionic), sulfate (anionic), quaternary ammonium salts (cationic) or betaines (zwitterionic). The hydrophobic part of siloxane surfactants comprises permethylated siloxane groups, e.g., a polysiloxane chain, trisiloxane or cyclotetrasiloxane [45]. An example of a siloxane surfactant is polydimethylsiloxane grafted with poly(ethylene oxide) (Figure 7).

**Figure 8:**
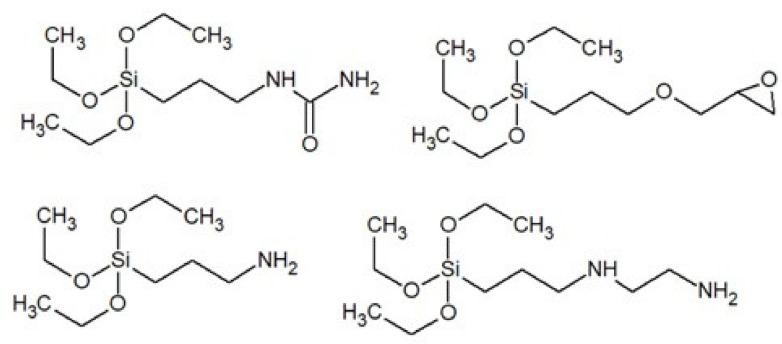
Examples of alkoxysilanes used in silicones.

In the silicone industry, silica (SiO_2_) is a popular filler. To improve the interaction of the inorganic filler with polysiloxane, alkoxy-functional silanes (dispersion stabilizers) are used [46]. Compounds of this type usually have in their structure three alkoxy groups attached to a silicon atom and a fourth group in the form of an aliphatic chain functionalized with amino, epoxy, carbamic groups, etc. (Figure 8).

**Figure 9:**
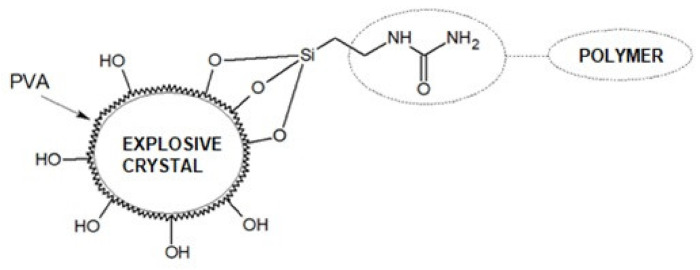
Explosive crystal modified with poly(vinyl alcohol) (PVA) and alkoxysilane dispersion stabilizer (scheme).

Alkoxy groups react with hydroxyl groups on the silica surface to form oxygen bridges (the by-product being alcohol), while the functionalized aliphatic group interacts with polysiloxane. Of course, on the surface of the explosive crystals, there are no hydroxyl groups capable of reacting with alkoxysilanes. The solution to this problem may be coating crystals with a layer of a substance with hydroxyl groups, e.g., poly(vinyl alcohol) (PVA, Figure 9).

**Figure 10:**
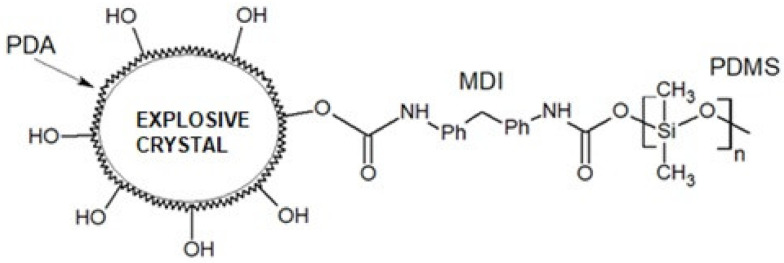
Explosive crystal modified with polydopamine (PDA)-g-PDMS (scheme).

The authors of [47] conducted similar tests on the functionalization of explosive crystals’ surfaces using polydopamine (PDA). The functional groups derived from polydopamine were then used to make grafted copolymers with other polymers: poly(glycidyl azide) (GAP), poly(ethylene glycol) (PEG) and polytetramethylene ether glycol (PTMEG). The polymers mentioned could be replaced by, e.g., polydimethylsiloxane terminated with hydroxyl groups (Figure 10). Isocyanates such as 1,1′-methylenebis(4-isocyanatobenzene) (MDI), 2,4-diisocyanato-1-methylbenzene (TDI) and 1,6-diisocyanatohexane (HDI), which are commonly used in the production of polyurethanes [48], can act as a link between polymers.

## 4. Conclusions

Polysiloxanes are an interesting group of polymers whose potential use in explosive compositions has already been considered in the past. Due to their very low glass transition temperatures, high thermal resistance and low reactivity, they would make a very good component for PBXs. The obstacle to the wider use of silicones in explosive compositions is their low affinity for explosive crystals. However, this is a problem that can be solved using simple steps. One possible solution is the functionalization of the explosive crystal’s surface. Polysiloxanes are not devoid of drawback compounds, but studies devoted to eliminating their negative properties may eventually lead to interesting results.

## Figures and Tables

**Table 1:** Physical properties of polydimethylsiloxane (PDMS) depending on the average molecular weight [14].

Average Molecular Mass [g/mol]	Dynamic Viscosity (25 °C) [mPa·s]	Dynamic Viscosity (25 °C) [mPa·s]	Surface Tension [mN/m]
600	3	0.90	19.3
1800	10	0.94	19.9
5800	100	0.97	20.9
26,000	1000	0.97	21.2
62,000	12,500	0.97	21.4
160,000	500,000	0.97	21.5

**Table 2:** Surface properties of selected polysiloxanes.

Polymer	Dynamic Viscosity (25 °C) [mPa·s]	Dynamic Viscosity (25 °C) [mPa·s]	Surface Tension [mN/m]
PDMS	20.7 [20]	22.7 [20]	23.5 [19]; 22.8 [20]
PMTFPS	24.2 [20]	21.4 [20]	13.6 [19,20]
PMPS	-	-	33.2 [19]
PMFPhS	-	-	18.7 [19]
PMDFPhS	-	-	19.1 [19]

**Table 3:** Glass transition temperatures of selected polysiloxanes.

Polymer	Glass Transition Temperature [°C]
PDMS	–123
PMHS	–138
PDES	–139
PMPS	–28
PDPS	40
PMTFPS	–70
PAPMS	–63

**Table 4:** Comparison of properties of plastic/polymer-bonded explosives (PBXs) with popular binders and PDMS [35,37].

Explosive	Loading Density [g/cm^3^]	Detonation Velocity [m/s]	Impact Sensitivity [J]	Friction Sensitivity [N]
RDX	1.76	8750	5.58	120
HMX	1.90	9100	6.37	95
BCHMX	1.79	8700	2.98	88
HNIW	2.04	9800	4.2	64
RDX/Viton A (91/9)	1.76	8285	10.6	326
HMX/Viton A (91/9)	1.84	8602	10.3	304
BCHMX/Viton A (91/9)	1.81	8474	5.3	283
HNIW/Viton A (91/9)	1.94	9023	6.9	252
RDX/Viton A (95/5)	1.76	8424	9.2	271
HMX/Viton A (95/5)	1.84	8730	9.2	243
BCHMX/Viton A (95/5)	1.81	8612	4.8	230
HNIW/Viton A (95/5)	1.95	9194	6.4	186
RDX/PDMS	1.58	7844	33.9	254
HMX/PDMS	1.64	8083	32.2	228
BCHMX/PDMS	1.63	7994	24.3	232
HNIW/PDMS	1.74	8267	28.0	192
RDX/HTPB	1.52	7526	14.6	>360
HMX/HTPB	1.57	7812	15.2	>360
BCHMX/HTPB	1.56	7746	9.6	322
HNIW/HTPB	1.63	8167	10.8	214

## Data Availability

Not applicable.
